# Supplementary material for: The microwave phase locking in Bloch transistor
Source: Nat Commun. 2026 Feb 2;17:1264. doi: 10.1038/s41467-025-67735-z (PMC12868743; doi:10.1038/s41467-025-67735-z)
Supplement: Supplementary file 1 — Supplementary Information [file 41467_2025_67735_MOESM1_ESM.pdf]

Supplementary Information for "The microwave phase locking in Bloch transistor"

Ilya Antonov[1], Rais S. Shaikhaidarov[1,2], Kyung Ho Kim[1,3], Dmitry Golubev[4,5], Sven Linzen[6], Evgeni V Il'ichev[6], Vladimir N Antonov[1], Oleg V Astafiev[1,7]

[1*]Physics, Royal Holloway University of London, Egham, TW20 0PN, Surrey, UK.
[2]National Physical Laboratory, Hampton Road, Teddington, TW11 0LW, UK.
[3]Department of Physics and Astronomy, Sejong University, Seoul, 05006, South Korea.
[4]HQS Quantum Simulations GmbH, Rintheimer Str. 23, Karlsruhe, 76131, Germany.
[5]Department of Applied Physics, QTF Centre of Excellence, Aalto, 610101, Finland.
[6]Leibniz Institute of Photonic Technology, Jena, D-07702, Germany.
[7]Skolkovo Institute of Science and Technology, Bolshoy Boulevard 30, Moscow, 121205, Russia.

Supplementary Note 1: Screening circuit of the Bloch transistor

The Bloch transistor requires five electrodes for the operation: current and voltage leads ($I+, I-, V+, V-$) and the gate electrode G, see Fig. S1. The JJs are screened from the environmental EM with TiN inductances, $L1 + L2 \sim 1.5\ \mu$H, and Pd resistors 6.3 kΩ.

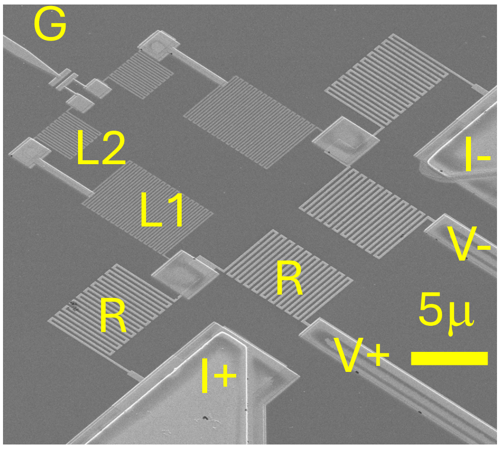

Fig. S1 Focused Ion Beam image of the BT with the screening circuit. The JJs of the BT are isolated from the environment by the circuit with TiN inductances $L1 + L2 \sim 1\ \mu$H and Pd resistors of 6.3 kΩ. The screening circuit is symmetric with respect to the JJs

Supplementary Note 2: *dc* symmetric amplifier

For the *dc* measurement we use a symmetric bias scheme with three instrumental amplifiers, see Fig. S2 [1]. There are two bias resistors $R_b = 100\,\mathrm{k}\Omega$ in each arm of the scheme. The voltage across the sample is taken at "out1" with an amplification factor G_1, while the current, I, is calculated from the voltage $2G_3R_bI$ at "out3". When sample resistance is high the scheme operates in the voltage bias regime , $R \gg R_b$, and in the current bias regime in the opposite case. At the current plateau we have $R \sim R_b$. This implies that the scheme is in an intermediate regime. It allows us to measure the back-bending in the $I - V$ curves.

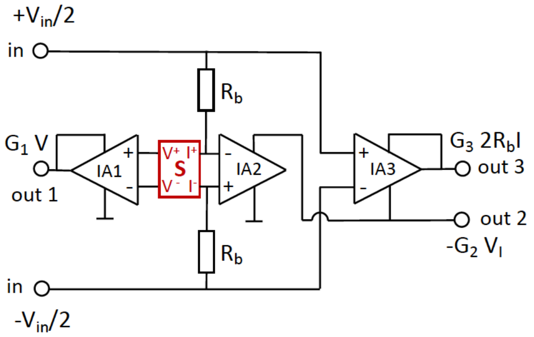

Fig. S2 The *dc* fully symmetric measurement circuit. The picture of circuit is taken from the Supplementary Information of [1].

Supplementary Note 3: The model of phase-locking in the Bloch transistor

The model of the phase-locking with the microwave (MW) in Bloch Transistor (BT) is based on the formalism developed by Averin, Zorin, and Likharev in [2]. We assume that the *ac* MW signal is applied to the gate electrode, see Fig. S3, so that one can approximately describe the system by the equation

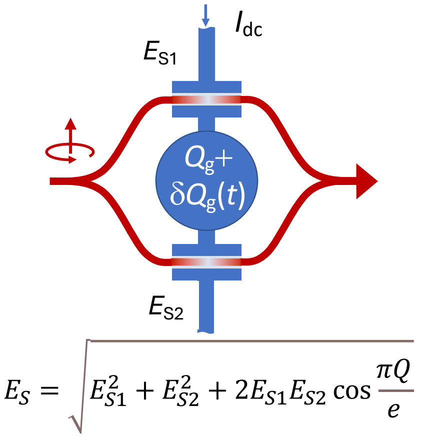

Fig. S3 Model of the phase-locking in the Bloch transistor. The charge $Q_{\rm g} + \delta Q_{\rm g}(t)$ is induced at the island by the gate voltage and MW. The $\delta Q_{\rm g}(t)$ is phase locked with the Bloch oscillations resulting in the quantized current, while Q_g modulates this quantization by means of E_S.

$$L\ddot{q} + R\dot{q} + V_{C_1}\sin\left(\frac{\pi q}{e} + \frac{\pi Q_g}{2e} + \frac{\pi \delta Q_g}{2e}\cos\omega t\right) + V_{C_2}\sin\left(\frac{\pi q}{e} - \frac{\pi Q_g}{2e} - \frac{\pi \delta Q_g}{2e}\cos\omega t\right) = V_b + \xi(t), \tag{S1}$$

where the terms proportional to V_{C_1} and V_{C_2} are the critical voltage of junctions 1 and 2, and $\xi(t)$ is the noise of the resistor. The critical voltage is related to the phase slip energy by $V_{C_1,C_2} = \pi E_{S_1,S_2}/e$. Here we assumed that up to time t the charge $q_1 = q + Q_g/2 + \delta Q_g\cos\omega t/2 + 2en$ has flown through junction 1 and the charge $q_2 = q - Q_g/2 - \delta Q_g\cos\omega t/2$ has flown through junction 2. These forms of transmitted charges are consistent with the condition that the central island can host only an integer number of Cooper pairs, which is explicitly read $q_1 - q_2 - Q_g(t) = 2en$. The term $2en$ drops out from Eq. (1) due to periodicity of the sine function. In the zeroth order of the perturbation theory we put $V_{C_1} = V_{C_2} = 0$ and find zeroth order solution for the charge

$$q_0(t) = \frac{V_b}{R}t + \frac{1}{R}\int_0^t dt'\left(1 - e^{-R(t-t')/L}\right)\xi(t'). \tag{S2}$$

In the next iteration we substitute this solution in the argument of sine functions in Eq. (S2) and solve the equation again. This results in

$$q(t) \; = \; q_0(t) - \delta q(t),$$

$$\delta q(t) \; = \; \frac{1}{R}\int_0^t dt' \left(1 - e^{-R(t-t')/L}\right) \times \left[V_{C_1} \sin\left(\frac{\pi q_0(t')}{e} + \frac{\pi Q_g}{2e} + \frac{\pi \delta Q_g}{2e}\cos\omega t'\right)\right. \quad \text{(S3)}$$

$$\left. + \, \mathrm{V}_{C_2} \sin\left(\tfrac{\pi q_0(t')}{e} - \tfrac{\pi Q_g}{2e} - \tfrac{\pi\delta Q_g}{2e}\cos\omega t'\right)\right].$$

We make one more iteration and substitute this solution in Eq. (S2) once again. However, this time we average the resulting equation over time and noise and find the average current flowing through the system $I_{\mathrm{dc}} = \langle \dot{q} \rangle$. In this way, we obtain the I-V curve in the form

$$I_{\mathrm{dc}} = \frac{V_b}{R} - \frac{1}{R}\left\langle V_{C_1} \sin\left(\frac{\pi[q_0(t) - \delta q(t)]}{e} + \frac{\pi Q_g}{2e} + \frac{\pi\delta Q_g}{2e}\cos\omega t\right)\right. \quad \text{(S4)}$$

$$\left. + \, \mathrm{V}_{C_2} \sin\left(\tfrac{\pi[q_0(t) - \delta q(t)]}{e} - \tfrac{\pi Q_g}{2e} - \tfrac{\pi\delta Q_g}{2e}\cos\omega t\right)\right\rangle$$

Now we assume that the critical voltages V_{C_1}, V_{C_2} are small. Then the charge fluctuations (S4) are also small, and we can make an expansion in Eq. (S5) in this parameter. The I-V curve takes the form

$$I_{\mathrm{dc}} \; = \; \frac{V_b}{R} + \frac{\pi}{eR}\left\langle \left[V_{C_1}\cos\left(\frac{\pi q_0(t)}{e} + \frac{\pi Q_g}{2e} + \frac{\pi\delta Q_g}{2e}\cos\omega t\right)\right.\right. \quad \text{(S5)}$$

$$\left.\left. + \, \mathrm{V}_{C_2}\cos\left(\tfrac{\pi q_0(t)}{e} - \tfrac{\pi Q_g}{2e} - \tfrac{\pi\delta Q_g}{2e}\cos\omega t\right)\right]\delta q(t)\right\rangle \; = \; \tfrac{V_b}{R} + \tfrac{\pi}{eR^2}\int_0^t dt' \left(1 - e^{-R(t-t')/L}\right)$$

$$\times \left\langle \left[V_{C_1}\cos\left(\tfrac{\pi q_0(t)}{e} + \tfrac{\pi Q_g}{2e} + \tfrac{\pi\delta Q_g}{2e}\cos\omega t\right) + V_{C_2}\cos\left(\tfrac{\pi q_0(t)}{e} - \tfrac{\pi Q_g}{2e} - \tfrac{\pi\delta Q_g}{2e}\cos\omega t\right)\right]\right.$$

$$\left. \times \left[V_{C_1}\sin\left(\tfrac{\pi q_0(t')}{e} + \tfrac{\pi Q_g}{2e} + \tfrac{\pi\delta Q_g}{2e}\cos\omega t'\right) + V_{C_2}\sin\left(\tfrac{\pi q_0(t')}{e} - \tfrac{\pi Q_g}{2e} - \tfrac{\pi\delta Q_g}{2e}\cos\omega t'\right)\right]\right\rangle.$$

Next, we use the property of Bessel functions, $e^{iz\cos\theta} = \sum_n i^n J_n(z) e^{in\theta}$, and transform the previous equation to the form

$$I_{\mathrm{dc}} \; = \; \frac{V_b}{R} - \frac{\pi}{2eR^2}\sum_n J_n^2\left(\frac{\pi\delta Q_g}{2e}\right)\int_0^t dt' \left(1 - e^{-R(t-t')/L}\right) e^{-\frac{\pi^2}{2e^2}\langle[q_0(t) - q_0(t')]^2\rangle} \quad \text{(S6)}$$

$$\times \sin\left[\left(\frac{\pi V_b}{eR} - n\omega\right)(t-t')\right] \times \left[V_{C_1}^2 + V_{C_2}^2 + (-1)^n 2V_{C_1}V_{C_2}\cos\frac{\pi Q_g}{e}\right].$$

Since V_{C_1}, V_{C_2} are small, we can replace $V_b \to I_{\rm dc}R$. Subsequently, we re-write the I-V curve in terms of the voltage drop on the two junctions $V = V_b - I_{\rm dc}R$,

$$\begin{aligned} V &= \frac{\pi}{2eR}\left[V_{C_1}^2 + V_{C_2}^2 + 2V_{C_1}V_{C_2}\cos\frac{\pi Q_g}{e}\right]\sum_n J_n^2\left(\frac{\pi\delta Q_g}{2e}\right) \\ &\times \int_0^\infty dt\left(1 - e^{-Rt/L}\right)e^{-\frac{\pi^2}{2e^2}\langle[q_0(t)-q_0(t')]^2\rangle}\sin\left[\left(\frac{\pi I_{\rm dc}}{e} - n\omega\right)t\right]. \end{aligned} \tag{S7}$$

This expression can be written in the form

$$V(I_{\rm dc}) = \sum_n J_n^2\left(\frac{\pi\delta Q_g}{2e}\right)V_0(I_{\rm dc} - 2efn), \tag{S8}$$

where the I-V curve in the absence of the microwave signal is

$$V_0(I_{\rm dc}) = \frac{\pi}{2eR}\left[V_{C_1}^2 + V_{C_2}^2 + 2V_{C_1}V_{C_2}\cos\frac{\pi Q_g}{e}\right]\int_0^\infty dt\left(1 - e^{-Rt/L}\right) \times e^{-\frac{\pi^2}{2e^2}\langle[q_0(t)-q_0(t')]^2\rangle}\sin\frac{\pi I_{\rm dc}t}{e}. \tag{S9}$$

Taking the derivative of Eq. (S8), we obtain the differential resistance at non-zero microwave power in the form

$$\frac{dV(I_{\rm dc})}{dI_{dc}} = \sum_n J_n^2\left(\frac{\pi\delta Q_g}{2e}\right)\frac{dV_0(I_{\rm dc} - 2efn)}{dI_{dc}}, \tag{S10}$$

The slope of the quantized current increases and the width of the peak of the differential resistance broadens when the thermal current δI_T excited in the normal Pd resistor R is considered [3]

$$\delta I_T = \delta I_T(0)(1 + \alpha i_{ac}^2)^{1/10} \tag{S11}$$

$$I_T(0) = \frac{\pi k_B T}{2eR} \tag{S12}$$

$$\alpha = \frac{2e^2 f^2 R}{\Sigma\nu T^5}, \tag{S13}$$

where $\Sigma = 1.2\times10^9$ W K^{-5} m^{-3} is the material constant of palladium [4], $\nu = 0.315\ \mu$m^3 is the volume of a single resistor, and $i_{ac} = I_{ac}/2ef$ is the normalized amplitude of the current induced in the resistor by MW.

Following the previous calculations, one can also introduce the phase slip energies of the BT E_S:

$$E_S = \sqrt{E_{S_1}^2 + E_{S_2}^2 + 2E_{S_1}E_{S_2}\cos\frac{\pi Q_g}{e}}. \tag{S14}$$

Supplementary Note 4: Parameters of the co-fabricated JJ

Fig. S4 shows the $I - V$ curve of the co-fabricated double Josephson junction with sizes 40×100 nm^2, which is not integrated into the BT screening circuit. The JJ has a critical current I_C ∼105 nA, exceeding the apparent current of the BT by almost one order of magnitude. The normal resistance of an individual junction is R_N= 1.5 kΩ.

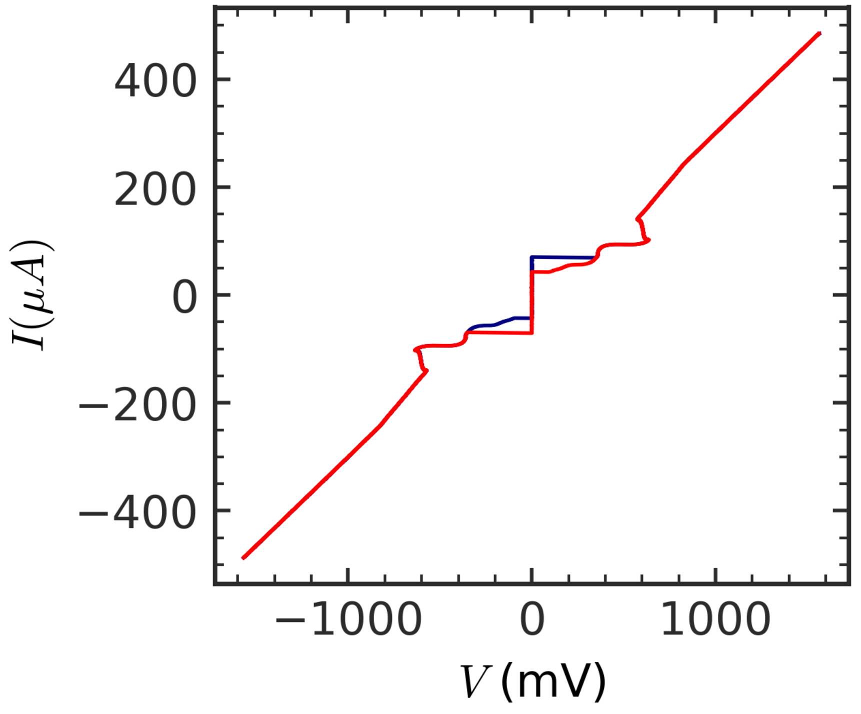

Fig. S4 $I - V$ curve of the double JJs co-fabricated with the BT. The critical current and normal resistance of the individual junction are I_C=105 nA and R_N= 1.5 kΩ respectively. The red and blue curve are two directions of the current sweep during the measurements.

Supplementary Note 5: Current quantization at different frequencies and gate voltages

We compile the $I-V$ curves showing current quantization at different frequencies in Fig. S5.

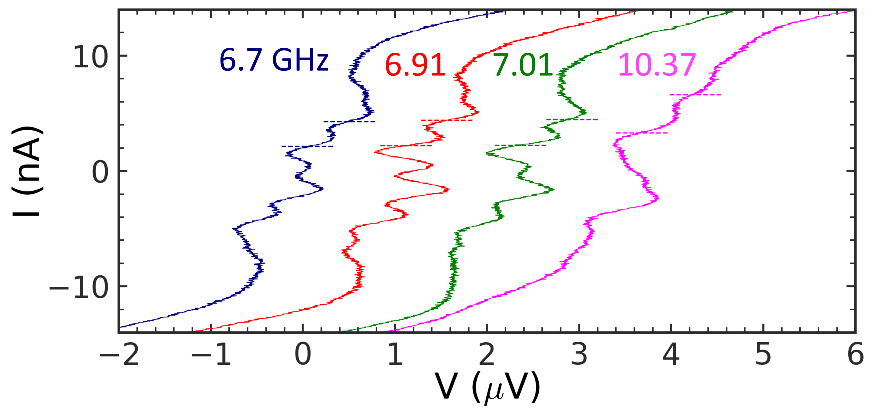

Fig. S5 Current quantization at different frequencies

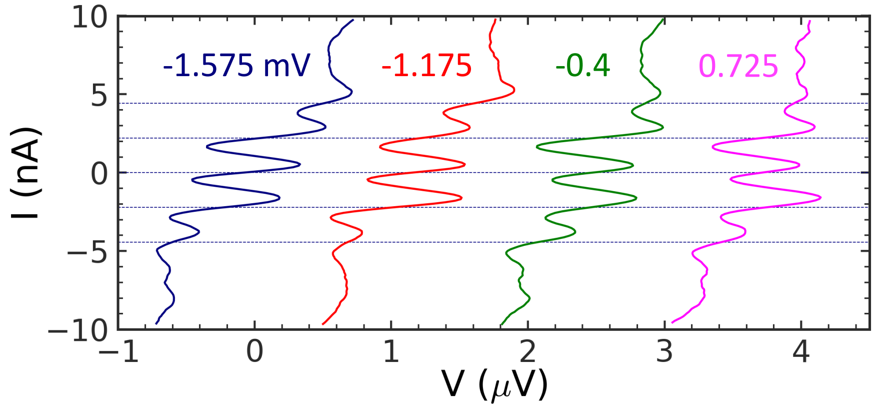

Fig. S6 Current quantization at different gate voltages. The MW frequency is fixed to 6.91 GHz. The $I-V$ curves are obtained by numerical integration of the experimental differential resistance.

Supplementary Note 6: Coulomb Blockade Oscillations of the Single Electron Transistor

The period of gate modulation of the BT current, ΔV_g, can correspond to charge e ($\Delta V_g = e/C_g$), or $2e$ ($\Delta V_g = 2e/C_g$) depending on the presence of quasi-particles in the superconductor. An independent measurement is required to distinguish between these cases. One can decide on the modulation period by

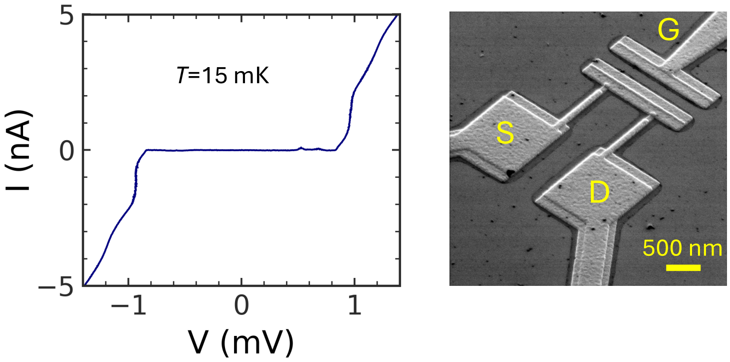

Fig. S7 SET with the lateral sizes identical to BT. **(Left)** $I-V$ curve of SET taken at 15 mK. **(Right)** Focused Ion Beam image of the SET. The area of the JJs, 40×80 nm^2, is close to that of BT.

measurement of the Single Electron Transistor (SET) with geometry identical to the BT. Since the gate capacitances are the same, the period of Coulomb Blockade Oscillations (CBO) in SET can give a reference. It is known that in the SET, the period of

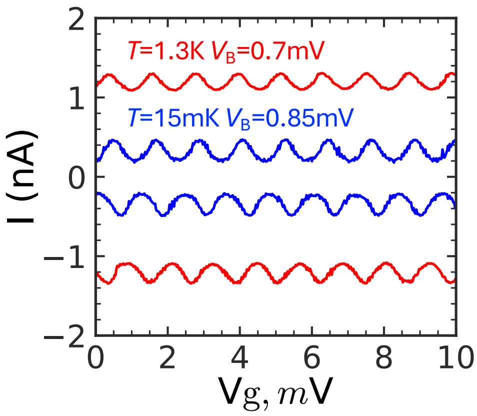

Fig. S8 Coloumb blockade oscillations of the SET at four different bias voltages, $V_b = \pm$ 0.7 mV and $\pm$ 0.85 mV, and two temperatures, $T =$ 15 mK and 1.3 K.

CBO corresponds predominantly to the charge of an electron e [5]. We measure the CBO of this Al SET. The photo and $I-V$ curve of the SET are shown in Fig. S7. The JJs have

normal resistance R_N= 188 kΩ, so that $E_C/E_J \sim 75 \gg 1$. The SET is in a well-defined charge state, so the current is blocked until the bias voltage exceeds $E_C/2e$. The period of CBO is $\Delta V_g \simeq$1.1928 mV, which determines C_g=0.134 fF, see Fig. S8. The period of dV/dI modulation in BT is very close, $\Delta V_g \simeq$1.1933 mV, confirming that the period corresponds to charge e.

Supplementary Note 7: Effect of the charge noise on the BT

The control of the BT with the gate voltage has a peculiarity: in fresh samples, the phase of the dV/dI modulation shows arbitrary jumps (Fig. S9,left). We attribute this effect to fluctuating substrate charge, which smears the Aharonov-Casher effect. Annealing the sample at 4 K, followed by cooling to 15 mK, suppresses these fluctuations (Fig. S9, right). Fig.3 of the main text was compiled after annealing.

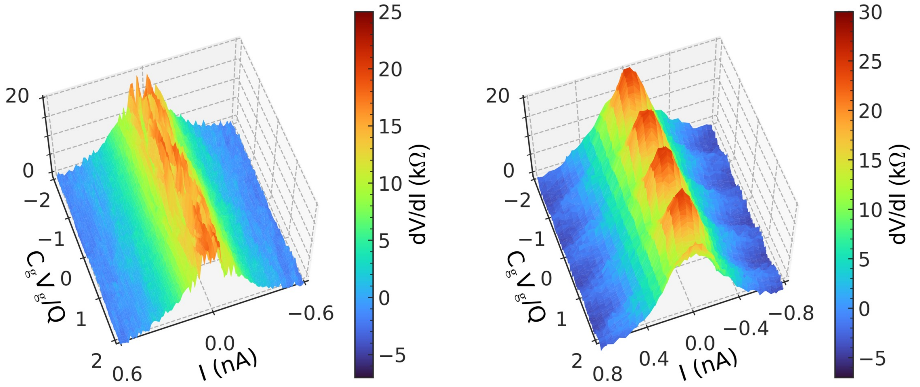

Fig. S9 **(Left)** The gate dependence of the BT differential resistance dV/dI in a fresh sample. The arbitrary phase jumps obscure the periodic Aharonov-Casher oscillations. **(Right)** The gate dependence after annealing BT at 4 K. The MW in both experiments is switched off.

Supplementary References

[1] R. S. Shaikhaidarov, K. H. Kim, J. Dunstan, I. Antonov, D. Golubev, V. N. Antonov, and O. V. Astafiev, Nature Communications **15**, 9326 (2024).

[2] D. Averin, A. Zorin, and K. Likharev, Sov. Phys. JETP **61**, 407 (1985).

[3] R. S. Shaikhaidarov, K. H. Kim, J. W. Dunstan, I. V. Antonov, S. Linzen, M. Ziegler, D. S. Golubev, V. N. Antonov, E. V. Il'ichev, and O. V. Astafiev, Nature **608**, 45 (2022).

[4] A. Vinante, P. Falferi, R. Mezzena, and M. Mück, Phys. Rev. B **75**, 104303 (2007).

[5] M. T. Tuominen, J. M. Hergenrother, T. S. Tighe, and M. Tinkham, Phys. Rev. Lett. **69**, 1997 (1992).
